# Potential role of gut-related factors in the pathology of cartilage in osteoarthritis

**DOI:** 10.3389/fnut.2024.1515806

**Published:** 2025-01-08

**Authors:** Peng Ning, Shuting Lin, Yongyan Shi, Tianjing Liu

**Affiliations:** ^1^Department of Pediatric Orthopaedics, Shengjing Hospital of China Medical University, Shenyang, China; ^2^Department of Pediatric Surgery, The First Affiliated Hospital of Guangxi Medical University, Nanning, China; ^3^Department of Pediatrics, Shengjing Hospital of China Medical University, Shenyang, China

**Keywords:** osteoarthritis, gut, microbiota, signaling pathway, molecular mechanism

## Abstract

Osteoarthritis (OA) is a common progressive degenerative disease. Gut microbiota (GM) and their metabolites have been closely associated with the onset, progression, and pathology of OA. GM and their metabolites may influence the cartilage directly, or indirectly by affecting the gut, the immune system, and the endocrine system. They function through classical pathways in cartilage metabolism and novel pathways that have recently been discovered. Some of them have been used as targets for the prevention and treatment of OA. The current study sought to describe the major pathological signaling pathways in OA chondrocytes and the potential role of gut-related factors in these pathways.

## Graphical Abstract

Gut-related factors in osteoarthritis (OA) include gut peptides, gut-derived hormones, and microbial metabolites and structures, all processed or influenced by the gut or gut microbiota. These factors impact several signaling pathways in chondrocytes, collectively regulating joint homeostasis. Some of these factors alleviate OA progression by playing positive roles in its pathogenesis, while others can exacerbate the disease. Additionally, some factors have uncertain roles. AMPK, AMP-activated protein kinase; BMP, bone morphogenic protein; CAT, capsiate; GLP-1, glucagon-like peptide 1; IPA, indole-3-propionic acid; MAPK, mitogen-activated protein kinase; mTOR, mammalian target of rapamycin; NF-κB, nuclear factor-kappa B; TGF, transforming growth factor. Created with BioRender.com. 
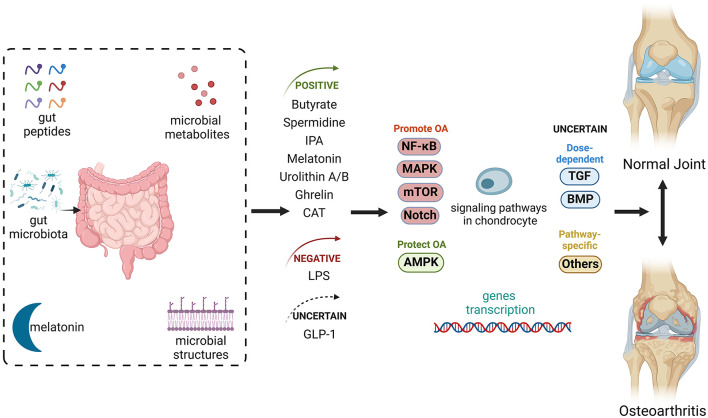

## 1 Introduction

Osteoarthritis (OA) is a low-grade inflammatory disease characterized by chronic articular cartilage degradation in joints such as hip and knee joints, thereby affecting the quality of life of patients (1). OA poses a substantial economic burden worldwide; however, the prevalence, incidence, and disability-free lifetime vary among countries and regions (2). Despite extensive research, the complex mechanisms underlying OA remain poorly understood, highlighting the need to identify key molecular pathways involved in its pathogenesis and to develop effective therapeutic strategies.

The intestinal tract harbors a vast variety of gut microbiota (GM), including viruses, fungi, and bacteria. GM disturbance, termed gut dysbiosis, refers to aberrant alterations in the diversity, composition, and function of GM. Dysbiosis has been linked to various types of arthritis, now collectively referred as the “gut-joint axis” accompanied with the gut-joint interaction. Compared with rheumatoid arthritis (RA) and spondyloarthritis (SpA), OA demonstrates distinct patterns of GM involvement due to differences in its underlying mechanisms. While RA and SpA are primarily driven by immune-mediated inflammation (3, 4), OA is characterized by chronic low-grade inflammation associated with obesity, metabolic syndrome, and diets rich in saturated fats, with GM acting as a key mediator (5).

In RA, gut-related pathogenesis is thought to originate at mucosal sites, driven by interactions between the mucosal immune system and altered local microbiota. Enhanced activation of Th17 cells, group 3 innate lymphoid cells, and mucosa-associated invariant T cells has been observed in RA patients (3). Similarly, SpA is closely associated with subclinical gut inflammation, with shared immunopathogenic pathways between the gut and joints. This is further validated by the clinical success of therapies targeting tumor necrosis factor and interleukin-23 in both inflammatory bowel disease and SpA

(4). In contrast, the role of GM in OA is more closely related to metabolic and inflammatory processes rather than direct immune activation. Dysbiosis contributes to chronic inflammation by altering gut permeability, promoting endotoxemia, and disrupting metabolic homeostasis, which collectively influence joint health.

Numerous studies have demonstrated that OA patients exhibit significant alterations in GM composition compared with healthy individuals, such as reduced alpha diversity and increased bacterial products like lipopolysaccharides (LPS). Higher abundances of *Clostridium, Streptococcus, Bacteroides*, and *Firmicutes* have been consistently observed in OA patients (6, 7). Interestingly, a large population-based cohort study identified associations between *Streptococcus* species and knee joint pain and inflammation (8), while the abundances of *Fusobacterium, Faecalibacterium*, and *Ruminococcaceae* were linked to histological OA severity and inflammatory biomarkers (9). These findings highlight the role of GM dysbiosis in amplifying chronic inflammation and its contributions to OA progression. Moreover, GM composition may vary depending on the OA site. For instance, a patient with symptomatic hand OA exhibited a low relative abundance of *Roseburia* but higher levels of *Bilophila* and *Desulfovibrio* (10).

In summary, while the “gut-joint axis” is a common theme across arthritis types, the specific contributions of GM vary considerably. RA and SpA are primarily immune-mediated, while OA involves metabolic and inflammatory pathways influenced by GM dysbiosis. Understanding these differences is crucial for developing GM-targeted interventions tailored to the unique pathogenesis of OA. This review explores the role of GM in OA pathogenesis, focusing on its influence on joint health and the potential molecular mechanisms.

## 2 Gut microbial metabolites, immune regulation, gut permeability, and microbiota translocation: mechanisms connecting the GM to OA

The human microbiome is defined as all microorganisms residing in the human body and their genes and products. The human gut harbors a large and dynamic microbial population (11). GM plays an important role in intestinal diseases such as inflammatory bowel diseases. In turn, intestinal inflammation aggravates gut dysbiosis by altering the gut environment (12). GM might contribute to extraintestinal diseases by converting and transmitting signals to the distal organs (13).

The association between GM and OA can be summarized in four parts: microbial metabolites (14, 15), immune regulation (16, 17), intestinal barrier (15, 18, 19), and microbiota translocation (20). Dysbiosis may affect gut permeability, which subsequently activates immune responses in joints owing to aberrant translocation of the microbiota and microbial metabolites into circulation, possibly inducing cartilage inflammation and destruction (21, 22).

### 2.1 Microbial metabolites

Intestinal metabolites are key actors in the gut-joint axis, as they are involved in various kinds of mechanisms and are also potential and promising intervention targets in OA. Several GM metabolites have been strongly implicated in the crosstalk between GM and OA.

#### 2.1.1 Short-chain fatty acid

Short-chain fatty acids (SCFAs), a large class of non-digestible carbohydrate-derived metabolites produced by GM, have been proven to be an important regulator of bone mass and bone homeostasis (23). Butyrate, one of the SCFAs, exerts chondroprotective effects against OA both *in vivo* and *in vitro*. Some studies have extensively explored the mechanism by which butyrate protects cartilage in OA. Butyrate reduces joint pain by inhibiting the signal transduction of pain at the dorsal root ganglion and down-regulates inflammatory cytokines and oxidative stress (24). Butyrate also down-regulates inflammatory cytokines/activity and up-regulates anti-inflammatory cytokines/activity (24–26). It also inhibits OA progression by decreasing inflammatory cell death and restoring the autophagic process in both chondrocytes (24, 27) and joint synovial tissue (24). In addition, butyrate increases the expression of Zonula occludens-1 (ZO-1) and occludin to enhance the intestinal tight junction (24).

#### 2.1.2 Bile acids

BAs are produced in the liver and metabolized by the GM in the intestine. Microbial modifications of BAs modulate host metabolism via BA receptors, such as the farnesoid X receptor (FXR) and the G-protein-coupled bile acid receptor Gpbar1 (TGR5) (28).

Some BAs, such as Tauroursodeoxycholic acid, may alleviate OA by preventing chondrocyte apoptosis (29) or promoting the restoration of OA cartilage (30). The former may decrease the apoptosis induced by increased endoplasmic reticulum stress (29), while the latter is related to the reduction in intracellular cholesterol levels and increase in membrane fluidity (30). However, all these results were obtained from *in vitro* cell cultures.

A previous study found that the expression of TGR5 decreased in human chondrocytes, and this could be reversed by its synthetic agonist INT 777, a semi-synthetic cholic acid derivative (31). Another study further investigated the possible mechanism and found that INT 777 inhibited the phosphorylation of p38 and the activation of the IκB kinase (IKK)/inhibitor of nuclear factor kappa B (IκBα)/nuclear factor-kappa B (NF-κB) signaling pathway to reduce collagen II and aggrecan degradation (32).

The role of BA has been extended beyond articular degenerative inflammation by the concept of “gut-muscle joint” (33). GM depletion disturbs microbial BA metabolism by increasing primary BAs, decreasing secondary BAs and inducing the production of FXR antagonists, which consequently inhibits the FXR-fibroblast growth factor (FGF) 15/19 signaling pathway in the gut to reduce circulatory FGF 15, causing skeletal muscle atrophy. Some scholars refined this concept as the “Gut microbiota-bile acid-skeletal muscle axis” (34).

#### 2.1.3 Tryptophan

Trp is an essential amino acid that can be processed by GM through direct transformation or the kynurenine pathway into metabolites such as indole, tryptamine, indole ethanol, indole-3-propionic acid (IPA), indole acetic acid (IAA), and indole-3-aldehyde (IAld) (35). Trp metabolites play an important role in microbiota-host crosstalk in healthy subjects and patients with metabolic and neuropsychiatric disorders (35). Trp metabolite disturbance is associated with OA. Rushing et al. (36) found that the composition of indole and its metabolites were particularly different in the feces of OA patients compared to healthy controls. Binvignat et al. (37) found that serum Trp metabolites were positively or negatively correlated with hand OA based on the type of Trp. The low level of plasma indole-3-lactic acid (ILA) was associated with symptomatic hand OA (38).

Dysbiosis may stimulate the expression of aryl hydrocarbon receptor (AhR), cytochrome P450 of family 1, subfamily A and polypeptide 1 (CyP1A1) in chondrocytes, thereby activating Trp synthesis and accelerating OA progression (39). However, Zhuang et al. (40, 41) discovered that IAld and IPA alleviated chondrocyte inflammation by downregulating AhR.

#### 2.1.4 Other metabolites

Spermidine is produced by intestinal bacteria and can enter the circulation via the colonic epithelium (42). It rescues dysregulated chondrocyte autophagy and prevents chondrocyte hypertrophy, contributing to cartilage maintenance (43). Spermidine can also enhance the autophagic flux by inducing autophagy proteins.

Urolithin is a GM metabolite that may protect against osteoporosis and OA. Urolithin A alleviates senile osteoporosis by enhancing the autophagy capacity of bone marrow macrophages (44) and relieves pain in OA by improving mitophagy and mitochondrial health (45). Similarly, urolithin B has been shown to inhibit inflammation, thereby reducing cartilage degeneration and ameliorating OA (46).

### 2.2 Immune regulation

GM activates immune cells that may migrate into the joints. Huang et al. first proposed a two-hit model of OA pathogenesis using LPS (47). They found that high serum LPS activated the innate immune, which may exacerbate the underlying or pre-OA pathology. Additionally, their study showed that the overloaded or injured joint synergistically activated innate immunity. Their later study further confirmed the association between serum LPS-binding protein (LBP) and the activated macrophages in the knee joint capsule and synovium (48). They also found that fecal microbiota transplantation from metabolically compromised patients to germ-free mice increased gut permeability accompanied by endotoxemia and systemic low-grade inflammation to aggravate OA severity. Overall, these findings suggest that GM exerts its immunomodulatory effects on OA by influencing the innate and adaptive immune response via regulation of immune cells such as macrophages and neutrophils. Macrophages were activated by LPS and associated with OA-related pain (48). Neutrophils work synergistically with macrophages in OA (49).

### 2.3 Gut permeability

The intestinal barrier, a multilayered defense system supplemented by numerous epithelial and non-epithelial cells, is divided into mucus, epithelium, and mucosal immune layers according to the defense mechanism (50). The gut-derived pathogen transport starts from a “leaky gut,” which acts as a key initiator of the gut-joint axis. It is the result of compromised intestinal barrier function and increased intestinal mucosal paracellular permeability (51). Intestinal barrier disruption allows opportunistic commensal and pathogenic microbes, as well as their products, to translocate across the intestinal barrier into the bloodstream, potentially triggering systemic infections (48). While increased intestinal permeability can lead to greater LPS exposure, the development of OA may occur independently of dysbiosis (8).

Under healthy conditions, bacteria and bacterial products are abundant in the outer mucus layer, which acts as a barrier between bacteria and epithelium. Commensal bacteria grow and form colonies in the outer mucus layer (52). However, dysbiosis may destruct the gut barrier in some conditions, which may be related to the progress of OA. For example, GM composition is an important regulatory factor of the intestinal mucus barrier function (53). GM can change the expression pattern of glycosyltransferases to influence the mucus barrier. Besides GM, microbial products such as LPS and flagellin can also bind to the promoters of mucin 2 to improve the mucus barrier by activating the NF-κB pathway at the transcriptional and epigenetic levels. GM and its metabolites such as butyrate and secondary BAs can regulate P-glycoprotein, an important component of the epithelium barrier (54).

The connection between GM, leaky gut, and OA has been verified by the “atopobiosis” phenomenon. The composition of GM changed with decreased expression of tight junction proteins ZO-1 and occludin in an animal model of OA (24). Bacterial nucleic acids were detected in the synovial tissues of OA patients. Patients with different types of arthritis pathology showed differences in microbiota composition (55). An exploratory study demonstrated the relationship between LPS and plasma microbiome in OA patients with obesity; however, whether the increased gut permeability is responsible for this relationship warrants further exploration (56). The effect of prebiotics on OA also supported the existence of leaky gut. Prebiotics alleviated cartilage degeneration, osteophyte formation, and inflammation in post-traumatic OA by promoting the expression of ZO-1 and occludin in the gut (57).

### 2.4 Microbiota translocation

Microbiota translocation, also known as atopobiosis, is the process by which bacteria or bacterial genetic materials cross the gut barrier to reach the joint via blood circulation (58). Besides blood circulation, lymphatic transport is also a possible route. Some lymphatics from the gut are connected to lymphatics of the sacroiliac joints and spine through the thoracic duct (59); however, the incidence of OA in these parts is lower than that in the knee (1).

Joint invasion by bacterial DNA or antigens might follow atopobiosis (59). The trained immunity can be induced after the accumulation of the microbial load to some extent. Nonetheless, whether viable bacteria persist in the joint for a long period remains controversial. Bacterial DNA patterns in the cartilage vary between the eroded and the intact area in one OA joint. The eroded area is more likely to expose to the products from systemic circulation, resulting in faster deposition and/or changes in bacterial DNA in the eroded area compared to those in the intact area (60).

The above hypothesis may be supported by the discovery of intra-articular microbes related to immunologic signatures and pathways (61) and some microbial products related to the activated macrophages in the synovium (8, 48).

## 3 Age, gender, and ethnicity may influence GM and OA

Age, gender, and ethnicity could influence GM composition and diversity, which in turn affect inflammation and immune responses crucial for OA pathogenesis. OA is more prevalent among women and the elderly, with ethnic differences observed in its incidence (1). A previous review summarized evidence linking systemic factors such as age, gender, obesity, and diet to GM's indirect role in OA (62). Age and gender may influence GM composition, abundance, diversity, and diurnal oscillation, contributing to age-related intestinal barrier dysfunction and GM-related hormonal changes that promote OA (62). Recent studies have reported an increased Firmicutes-to-Bacteroidetes (F/B) ratio in both women (63) and OA patients (6), with this trend being more pronounced in individuals over 50 years old (63). However, another case-control study found reduced energy metabolism and acetate production in elderly women with OA, accompanied by a decreased F/B ratio (64). Given the diversity within the *Firmicutes* and *Bacteroidetes phyla*, relying solely on the F/B ratio oversimplifies GM characteristics in OA. Further analysis showed that within the *Firmicutes* phylum, *Clostridium* increased in OA, aging, and women compared to men, while *Faecalibacterium* decreased in both OA and aging (64, 65).

Aged mice exhibit a higher ratio of primary to secondary bile acids, resembling changes in the “Gut microbiota-bile acid-skeletal muscle axis” mentioned in Section 2.1.2 (66). Interestingly, co-housing aged and young mice ameliorated age-related GM imbalances (66). Another study reported increased alpha diversity in cartilage microbiota with age and OA, contrasting with decreased alpha diversity in cecal microbiota under aging and high-fat diets (67).

Ethnic differences in OA exist but lack consensus. Studies on OA incidence by race have yielded inconsistent results across regions, with some U.S. based studies reporting higher rates among African Americans compared to whites (68). Ethnic differences also manifest in OA-related pain and function, influenced by factors such as psychological resilience, perceived stress, depressive symptoms, and low income (68). However, no direct evidence links ethnicity to OA through GM regulation.

## 4 Gut-related factors influence chondrocyte pathways in OA pathogenesis

Gut-related factors may work on the cartilage through various pathways, including NF-κB, mitogen-activated protein kinase (MAPK), Notch, AMP-activated protein kinase (AMPK), mammalian target of rapamycin (mTOR), and transforming growth factor-beta (TGF-β)/bone morphogenic protein (BMP) pathways. In this section, we will discuss the major ways in which GM affects the incidence and development of OA, as well as related pathways, and update on the potential therapeutic role of gut-related factors in the pathogenesis of OA in these pathways.

### 4.1 NF-κB pathway

#### 4.1.1 NF-κB pathway in OA

The NF-κB signaling pathway in OA has two aspects: the classical and the alternative pathways. Most gut-related factors function via the classical pathway.

According to the previous studies (69, 70), the classical NF-κB pathway is triggered when cytokines such as tumor necrosis factor-α (TNF-α), interleukin-1β (IL-1β), and LPS combine with T cell receptors, including the tumor necrosis factor receptor (TNFR), Toll-like receptor (TLR), and T cell receptor (TCR), thereby activating the inhibitor of kappa B kinase complex (IKKα/IKKβ/IKKγ-NEMO). Activated IKKs can cause ubiquitin (Ub)-proteasome-mediated degradation through IκB phosphorylation. The activated NF-κB dimer is transferred to the nucleus functioning on the target genes. NF-κB dimers of the classical pathway include RelA (p65), c-Rel, and NF-κB1 (p105, a precursor inactive form of p50/p50) subunits. Hypoxia-inducible factor-2α (HIF-2α) can enhance this activation, resulting in a stronger NF-κB pathway and severe joint inflammation.

The activated NF-κB pathway induces the synthesis of metalloproteases (MMPs), vascular endothelial growth factor (VEGF), osteocalcin, or a disintegrin and metalloproteinase with thrombospondin motifs (ADAMTS), causing hypertrophy in chondrocytes and destroying cartilage and synovial membranes (70, 71).

#### 4.1.2 Role of gut-related factors in the NF-κB pathway

*Flagella*, LPS, lipoteichoic acid, and bacterial cell wall peptidoglycan components are components of the microbiota structure and can induce inflammation via receptor pattern recognition receptors (PRRs) (72). An animal study found a higher level of LPS in the circulation and joint fluids, which resulted in a higher rate of OA and decreased cartilage thickness (73). Elevated expression of TLR4 and MMP-13 and accelerated cartilage degeneration were also observed. LPS exerts pathogenic effects on OA possibly by binding to TLR4, thereby increasing cytokine production by activating NF-κB, resulting in increased MMPs and decreased collagen and proteoglycan synthesis (25). LPS increases nuclear-phosphorylated p65 expression and decreases phosphorylated IκB-α expression in chondrocytes. Increased gut permeability induced by TLR4 over-activation allows more LPS to enter the circulation, further aggravating the development of OA (19). The cartilage of human OA and OA-susceptible mice contain more Gram-negative bacterial DNA (60). Intracellular adaptors contain tumor necrosis factor receptor-related factor 6 (TRAF6), interleukin-1-receptor associated kinases 1 (IRAK1), and myeloid differentiation factor 88 (MyD88) in LPS-induced TLR4 signaling activation, and MyD88 is critical for NF-κB-mediated inflammation (74).

Spermidine inhibits NF-κB pathway activation by inhibiting IκBα degradation and p65 nuclear translocation, which may subsequently rescue the autophagy deficiency (43).

Spermidine could alleviate osteoarthritis (OA) both directly, through its effects on chondrocytes, and indirectly, by acting on other cartilage-associated cells such as macrophages. In chondrocytes, spermidine inhibits NF-κB pathway activation by preventing IκBα degradation and p65 nuclear translocation (43, 75), primarily through its interaction with the AhR (75). This mechanism involves the reduction of chondrocyte inflammation and inflammation-associated pyroptosis (75), while also restoring the efficiency of autophagic flux (43). Additionally, spermidine suppresses RIP1 ubiquitination in the TNF-α-driven NF-κB/p65 inflammatory pathway in fibroblast-like synoviocytes, thereby decreasing the secretion of IL-6, IL-8, and TNF-α, which ultimately mitigates chondrocyte degeneration (76). Furthermore, spermidine inhibits the ERK/MAPK and p65/NF-κB signaling pathways in macrophages, promoting anabolic processes and suppressing catabolic activity in chondrocytes (77).

Sodium butyrate might ameliorate human chondrocyte inflammation by suppressing IL-1β-induced IKK phosphorylation, IκBα degradation, and p65 nuclear translocation to inhibit the expression of MMPs and ADAMTS (26). A subsequent study confirmed that its protective effects are mediated by the G protein-coupled receptor 43 (GPR43) receptor (25).

Trp metabolites, including IAld and IPA can resist IL-1β-induced chondrocyte inflammation by decreasing pro-inflammatory cytokines. They can reduce extracellular matrix (ECM) degradation and promote matrix synthesis through the AhR/NF-κB axis. Trp metabolites also inhibit the IL-1β-induced NF-κB pathway by inhibiting IKKβ, IκBα, and p65 phosphorylation via AhR (40, 41). Moreover, IAld inhibits nuclear translocation of p65 and IκBα degradation (40).

Melatonin is a hormone rhythmically secreted by mainly the pineal gland and other peripheral organs including the colon. *Lactobacillus reuteri* and *Escherichia coli* can promote the expression of a rate-limiting enzyme for melatonin (78). The major pathway of melatonin biosynthesis is related to the anabolism of Trp and serotonin (79). Melatonin protects against OA through NF-κB and TGF pathways (80). After activating sirtuin1 (SIRT1), melatonin inhibits the overexpression of the NF-κB pathway in IL-1β-induced chondrocytes to inhibit chondrocyte matrix degradation-related proteins, which is mechanically linked to the inhibition of IκBα and p65 phosphorylation and the IκBα degradation (80).

SIRT1, a histone deacetylase enzyme involved in nicotinamide adenine dinucleotide metabolism, plays a protective role in OA mainly by regulating the NF-κB pathway (70). SIRT1 mediates the protective effects of melatonin in OA (81–83). In chondrocytes, melatonin has been shown to upregulate SIRT1 expression and suppress the inositol-requiring enzyme 1-α/X-box binding protein 1/C/EBP homologous protein pathway, thereby reducing endoplasmic reticulum stress-mediated apoptosis (83). Another study reported that the protective effects of melatonin on chondrocyte mitochondria are mediated through SIRT1 and superoxide dismutase 2 (81). Beyond chondrocytes, melatonin also mitigates D-galactose-induced reductions in hyaluronic acid production in synovial membrane cells via SIRT1 signaling (82).

Urolithin A and urolithin B exhibit similar mechanisms in resisting IL-1β-stimulated cartilage damage by inhibiting the activation of NF-κB pathway (46, 84). Urolithin A achieves this by suppressing p65 phosphorylation, while urolithin B inhibits the phosphorylation of IκB-α, with both ultimately preventing p65 nuclear translocation.

Ghrelin, a gut peptide secreted in enteroendocrine cells, is regulated by SCFAs, amino acids, formyl peptides, LPS, and hydrogen sulfide (85). It reversed IL-1β-induced activation of the NF-κB pathway to alleviate OA by suppressing phosphorylated IkBα in chondrocytes (86), via its receptor, the Growth Hormone Secretagogue Receptor (GHSR) (87).

Glucagon-like peptide 1 (GLP-1) is a gut peptide, preventing OA by binding to its GLP-1 receptor (GLP-1R) expressed in different cells of the joint (88). Because of the rapid degradation of native human GLP-1, most of the existing evidence of its therapeutic effect on OA have been explored by researching on its analogs such as liraglutide and exenatide (88). GLP-1 and GLP-1 analogs exert their effects by binding to the GLP-1 receptor (GLP-1R) expressed in various joint tissues, including chondrocytes, macrophages, adipocytes, and osteocytes. Consequently, GLP-1R agonists can influence multiple joint tissues, such as chondrocytes in cartilage, macrophages in the synovial membrane, adipocytes in Hoffa's fat pad, and osteoblasts in bone tissue. However, the current focus is primarily on chondrocytes (89). Recently, the novel GLP-1R agonist dulaglutide demonstrated a protective effect against AGEs-induced degradation of type II collagen and aggrecan *in vitro* chondrocytes (90). This effect is mediated through the NF-κB pathway, with a reduction in the translocation of the p65 protein.

**Figure 1:**
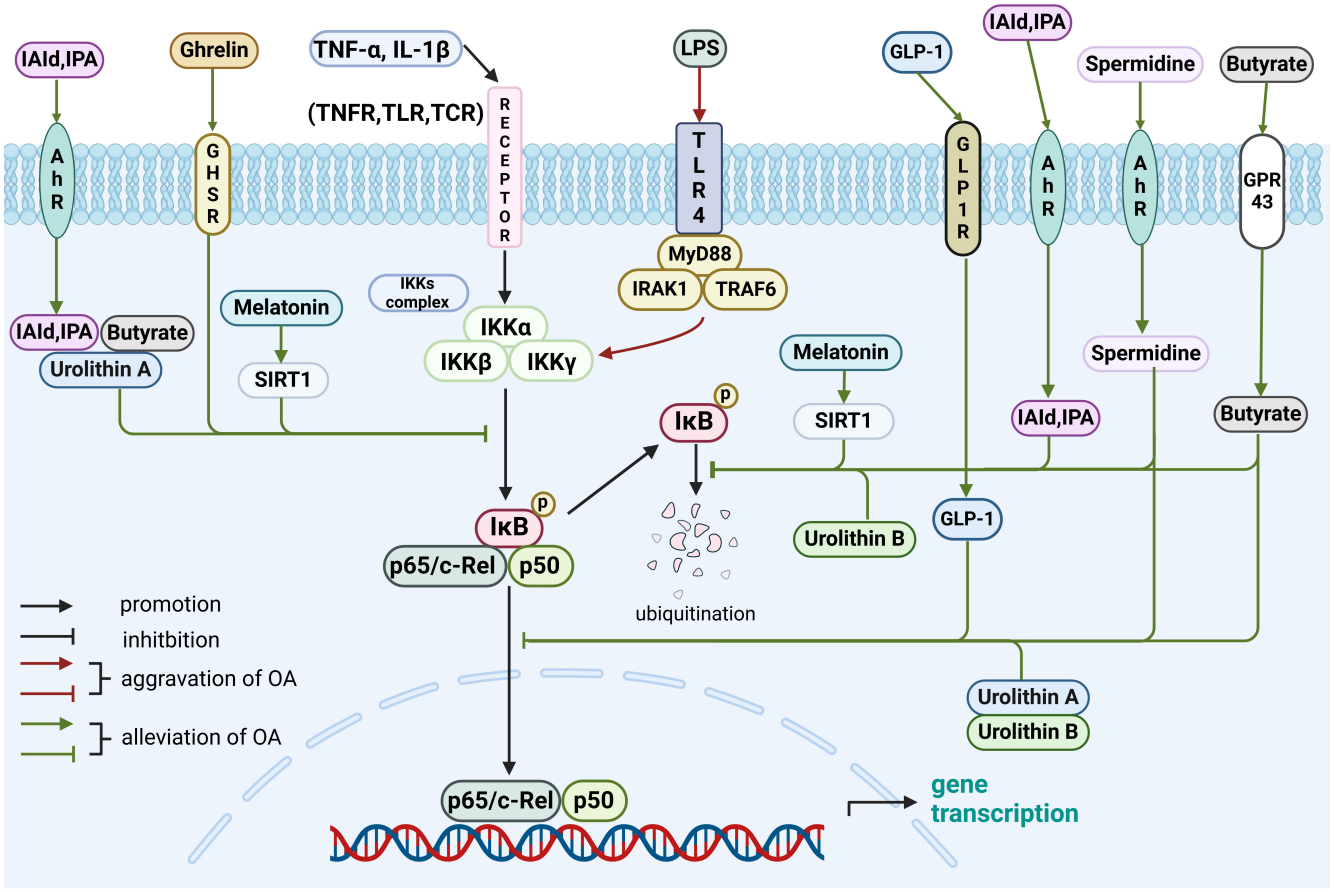
Role of gut-related factors in the pathogenesis of OA in the NF-κB pathway in chondrocytes. Gut-related factors: LPS, spermidine, butyrate, IAId, IPA, melatonin, urolithin A, urolithin B, ghrelin and GLP-1. AhR, aryl hydrocarbon receptor; GHSR, growth hormone secretagogue receptor; GLP-1, glucagon-like peptide 1; GLP-1R, GLP-1 receptor; GPR43, G protein-coupled receptor 43; IAld, indole-3-aldehyde; IκB, inhibitor of nuclear factor kappa B; IKK, IκB kinase; IL, interleukin; IPA, indole-3-propionic acid; IRAK1, interleukin-1-receptor associated kinases 1; LPS, lipopolysaccharide; MyD88, myeloid differentiation factor 88; SIRT1, sirtuin1; TCR, T cell receptor; TGF, transforming growth factor; TLR, toll-like receptor; TNF, tumor necrosis factor; TNFR, tumor necrosis factor receptor; TRAF6, tumor necrosis factor receptor-related factor 6. Created with BioRender.com.

All the gut-related factors in the NF-κB pathway are shown in Figure 1.

### 4.2 Notch pathway

#### 4.2.1 Notch pathway in OA

The Notch pathway plays a dual role in articular cartilage depending on temporal (91, 92) and spatial (91) factors. Persisting over-activation of the Notch pathway can disrupt joint homeostasis, which is partly responsible for cartilage degradation and OA progression (92–94).

In mammals, signaling translocation in the Notch pathway is activated by a short-term combination of receptors (Notch 1, 2, 3, and 4) and ligands (Delta-like 1, 3, and 4 and Jagged 1 and 2) from adjacent cells. The Notch pathway involves three processing cleavages: S1 cleavage represents the modification of the primary Notch protein by Furin-like convertase, forming mature Notch receptors. S2 cleavage entails the removal of the extracellular part of the combinator by ADAM10 or ADAM17/TNF-α converting enzyme (TACE). S3 cleavage is the modification of the residual part by γ-secretase to form the soluble Notch intracellular domain (NICD).

The key step is to transfer the NICD gained from receptor cleavages into the nucleus to interact with the transcription factor CBF-1/suppressor of hairless/Lag1 (CSL; C-repeat/DRE binding factor 1 (CBF1) in humans) (95). This is defined as canonical because of the requirement for cleavage and CSL dependence. The non-canonical pathway is CSL-independent via Abelson (Abl, a tyrosine kinase) murine leukemia viral oncogene homolog without cleavage (91). The Notch1,2-ADAM10-RBPJ-HES1 pathway is thus the major and concrete signaling in OA, with elevated MMPs and ADAMTS (93). Hairy and enhancer of split (HES) and recombination signal binding protein for Ig kappa J (RBPJ) are the downstream reaction factors.

#### 4.2.2 Role of gut-related factors in the notch pathway

Itchy E3 ubiquitin-protein ligase (ITCH), an E3 ubiquitin ligase, mitigates LPS-induced chondrocyte injury and OA-induced articular cartilage damage. Furthermore, LPS promotes Jagged 1 (JAG1) indirectly by suppressing the ITCH, possibly inhibiting JAG1 ubiquitination (96). JAG1 binds to the Notch receptor and releases NICD into the nucleus, which interacts with CSL to induce cellular inflammation, apoptosis, and ECM degradation.

The Notch pathway and TLR4 signaling synergistically mediate knee hyperalgesia in dorsal root ganglion (DRG) cells (97). The TLR4 signaling pathway activates the Notch pathway by increasing NICD. DRG tissue of donors with pain showed higher expression of HES1, JAG1, and RBPJa, supporting the role of the Notch pathway in OA pain.

**Figure 2:**
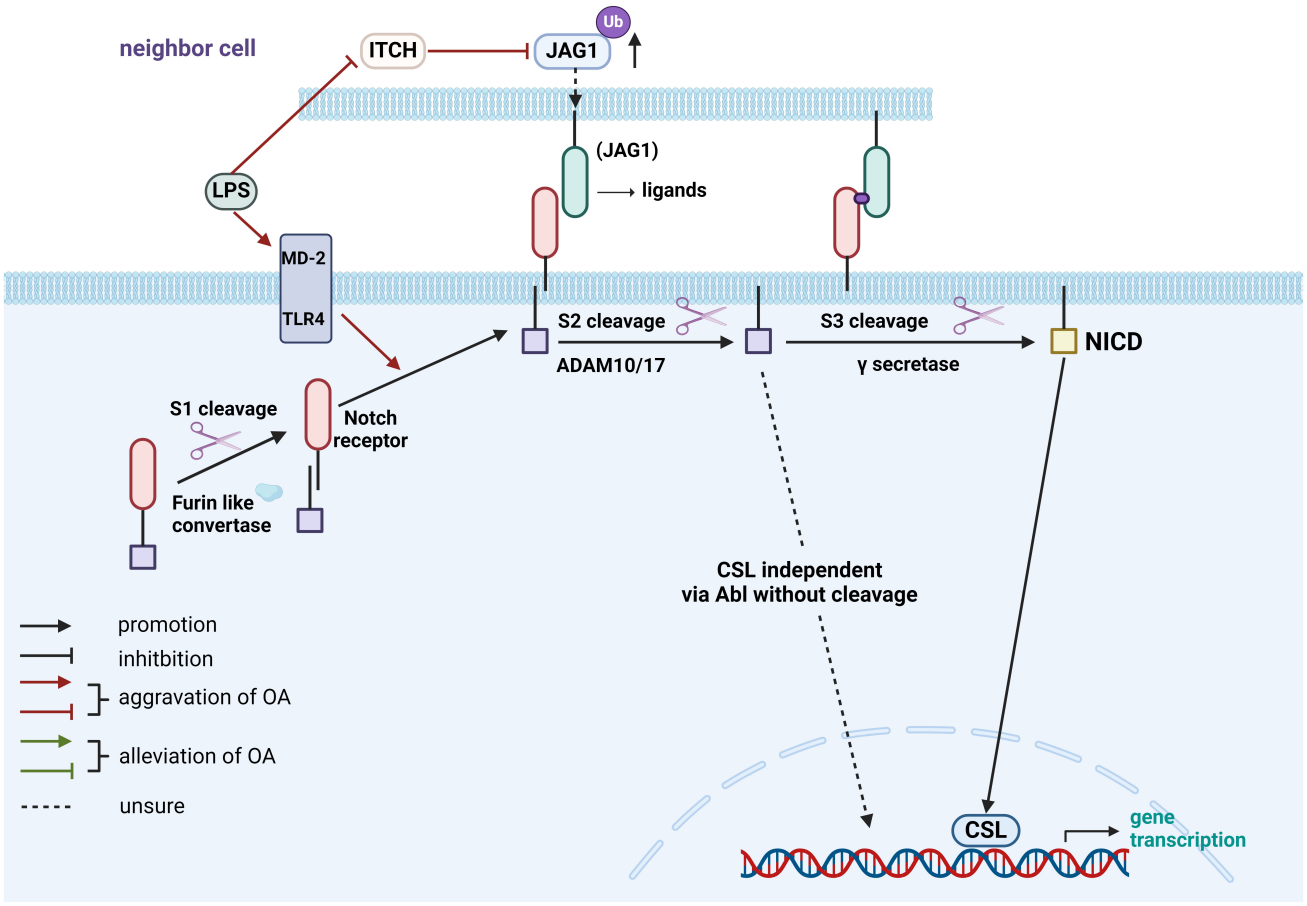
Role of gut-related factors in the pathogenesis of OA in the Notch pathway in chondrocytes. Gut-related factors: LPS. Abl, Abelson; ADAM, A Disintegrin and Metalloprotease; CSL, CBF-1/suppressor of hairless/Lag1; ITCH, Itchy E3 ubiquitin-protein ligase; JAG1, Jagged 1; LPS, lipopolysaccharide; MD-2, myeloid differentiation factor 2; NICD, Notch Intracellular Domain; TLR4, Toll-like receptor 4; Ub, ubiquitination. Created with BioRender.com.

LPS as a gut-related factor in the Notch pathway is shown in Figure 2.

### 4.3 The adenosine monophosphate-activated protein kinase and mechanistic target of rapamycin pathways

#### 4.3.1 The AMPK and mTOR pathways in OA

The pathogenesis of OA involves a complex interplay of factors, including the suppression of AMPK, activation of mTOR, and impaired autophagy within chondrocytes (98). Activation of AMPK-SIRT1/3 pathways improve the homeostasis of chondrocytes (99). The AMPK holoenzyme comprising α, β, and γ subunits can be phosphorylated by upstream kinases such as liver kinase B1 (LKB1), calcium/calmodulin-dependent protein kinase kinase-β (CaMKK-β), and TGFβ-activated kinase-1 (TAK1) (100). The AMPK pathway promotes autophagy and inhibits the activity of mTOR complex 1 (mTORC1) (101). In OA, AMPK phosphorylation is inhibited, and the SIRT expression is downregulated (98). Consequently, the downstream reactors peroxisome proliferator-activated receptor γ coactivator-1α (PGC-1α), and forkhead box class O 3a (FOXO3a) are also suppressed (99). The regulated in development and DNA damage response 1 (REDD1), an endogenous inhibitor of mTOR, can improve autophagy and mitochondrial biogenesis in articular cartilage and has been reported to enhance AMPK-induced PGC-1α transcriptional activation (102). SIRT1 may also indirectly suppress mTORC1. Numerous studies have reported that AMPK can inhibit the mTOR protein via the key regulator tuberous sclerosis complex 2 (TSC2) (103).

The AMPK- Unc-51 Like Autophagy Activating Kinase 1 (ULK1) axis plays a pivotal role in autophagy regulation. AMPK activation stimulates ULK1 phosphorylation, initiating autophagy while simultaneously inhibiting mTORC1. Conversely, mTORC1 can suppress autophagy by inhibiting both ULK1 and beclin-1 complexes. Additional signaling pathways, including phosphatidylinositol3-kinase/protein kinase B (PI3K/AKT; introduced in part 2.6.2), extracellular signal-regulated kinase (ERK)/MAPK, and peroxisome proliferator-activated receptor (PPAR), converge on mTOR, influencing its regulatory function (70, 98).

#### 4.3.2 Role of gut-related factors in the pathogenesis of OA in the AMPK-mTOR pathway

Butyrate can relieve OA pain, cartilage damage, and joint inflammation by modulating the gut environment and autophagic flux (24). Evidence suggests that it can reduce the IL-1β-induced phosphorylation of PI3K, AKT, and mTOR, thereby ameliorate OA via enhancing autophagy in chondrocytes (27). Melatonin activates AMPK and Foxo3 phosphorylation to stimulate autophagic flux and maintain the mitochondrial redox homeostasis in chondrocytes (104).

**Figure 3:**
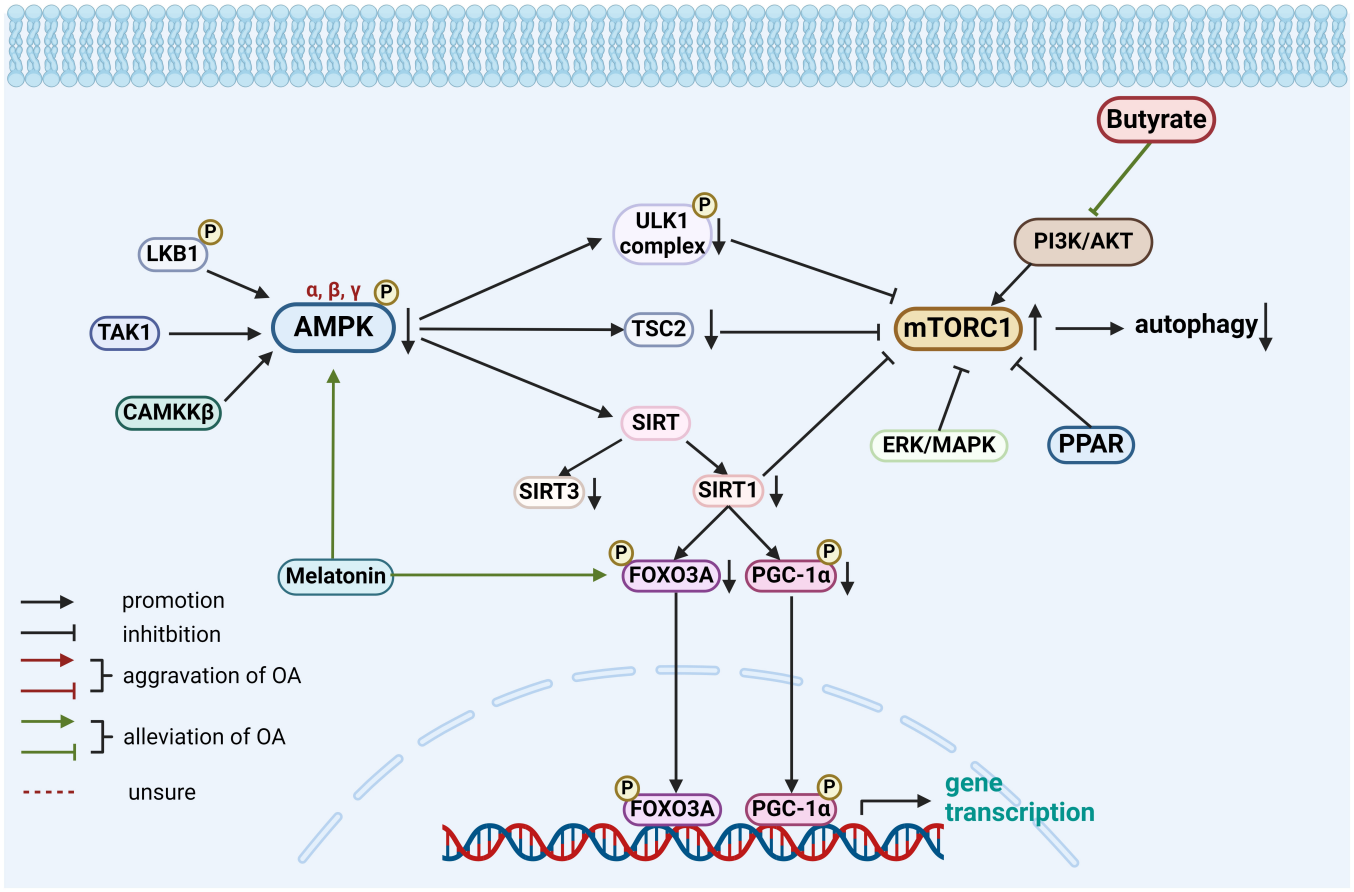
Role of gut-related factors in the pathogenesis of OA in the AMPK-mTOR pathway in chondrocytes. Gut-related factors: butyrate, melatonin. AMPK, AMP-activated protein kinase; CAMKK-β, calcium/calmodulin-dependent protein kinase kinase-β; ERK, extracellular signal-regulated kinase; FOXO3A, forkhead box class O 3a; LKB1, liver kinase B1; MAPK, mitogen-activated protein kinase; mTORC, mTOR complex; PGC-1α, peroxisome proliferator-activated receptor γ coactivator-1α; PI3K/AKT, phosphatidylinositol3-kinase/protein kinase B; PPAR, peroxisome proliferator-activated receptor; SIRT, sirtuin1; TAK1, TGFβ-activated kinase-1; TSC2, tuberous sclerosis complex 2; ULK1, Unc-51 Like Autophagy Activating Kinase 1. Created with BioRender.com.

The various gut-related factors associated with the AMPK-mTOR pathway are presented in Figure 3.

### 4.4 Transforming growth factor-β/bone morphogenetic protein pathway in OA and melatonin

The TGF-β superfamily comprises many members, including TGF-β and BMP. In TGF-β/BMP pathway initiates with ligand binding to a heteromeric complex of transmembrane serine/threonine kinase receptors. Type II receptors comprise TGFBRII and BMPRII, while type I receptors include activin-like kinase (ALK) 1-7. Specifically, TGF-β ligands bind to TGFBRII and ALK4, 5, or 7, whereas BMP ligands bind to BMPRII and ALK1, 2, 3, or 6 (70, 105, 106).

Similarly, the phosphorylation of receptor-regulated small mothers against decapentaplegic (R-Smad) proteins is induced by activation of receptors by their respective ligands. The phosphorylated R-Smad binds to Common partner Smad (Co-Smad) leading to the formation of a trimeric complex that enters the nucleus to regulate the transcription of target genes. The TGF-β signaling pathway can be simplified as: ligand binding to ALK5 receptor, followed by activation of Smad 2/3, which subsequently forms a complex with Co-Smad. In comparison, BMP signaling pathway involves ligand binding to ALK1 receptor, activating Smad 1/5/8, which then forms a complex with Co-Smad. In OA, the concentration of active TGF-β is elevated which leads to the activation of the ALK1-Smad1/5/8 pathway (105). Melatonin protects chondrocytes via activating the TGF-β1/Smad2 pathway and the upregulation of collagen II (80).

### 4.5 The mitogen-activated protein kinase pathway in OA and Urolithin A

In mammalian cells, primary functions of the MAPK pathway involve ERK, p38MAPK and c-Jun N-terminal kinase (JNK) (107), among which ERK1/2, p38MAPK, and JNK1/2 participate in the development of OA (108–110). The transduction of MAPK signal from outside into the nucleus occurs is driven by various phosphorylation cascades following activation of MAPK kinase kinases (MKKKs), MAPK kinases (MKKs), and MAPKs.

Activation of target genes upregulates MMPs and ADAMTS proteases while downregulating type II collagen, ultimately disrupting the normal cartilage extracellular matrix and contributing to cartilage degradation (108).

Urolithin A can inhibit the IL-1β-induced activation of the MAPK pathway by blocking ERK1/2, JNK, and p38 phosphorylation in a concentration-dependent manner (84). This results in attenuation of the IL-1β-induced degradation of collagen II and aggrecan and suppression of inflammatory mediators.

### 4.6 Other classical pathways and regulators in chondrocytes and the potential role of gut-related factors in these pathways

Besides the major relevant pathways mentioned above, several other signaling pathways and molecules have been found to be involved in the progression of OA. Here, we provide a brief summary of such molecules.

#### 4.6.1 Janus kinase/signal transducer and activator of transcription pathway in OA

Impaired JAK2/STAT3 pathway can induce pathological changes in OA, including cartilage destruction, subchondral bone destruction, and synovial inflammation (111). JAK phosphorylation triggers STAT recruitment, initiating subsequent gene transcription (112).

Ghrelin attenuates IL-1β-induced STAT3 phosphorylation, suppressing IRF-1 expression through the JAK2/STAT3 pathway, and ultimately reducing MMP and ADAMTS expression (113). Melatonin ameliorates OA by inhibiting the phosphorylation of JAK2 and STAT3 and suppress the levels of MMP-3, MMP-9, and MMP-13 as well as the NF-κB and TGF-β pathways (114).

Spermidine enhanced brahma-related gene 1 expression, which ameliorated the antioxidant ability of cartilage through the Nrf2 pathway and improved the inflammatory profile in OA cartilage by suppressing STAT3 phosphorylation (115).

#### 4.6.2 Phosphatidylinositol3-kinase/protein kinase B pathway in OA

Inhibiting the PI3K/AKT pathway can increase autophagy by blocking the mTOR activation in chondrocytes thereby alleviating OA (116–118). Similarly, activation of the PI3K/AKT/mTOR following overexpression of multiple EGF-like-domains 9 (MEGF9) may aggravate cartilage degradation (119). DAla2GIP, a gastric inhibitory polypeptide (GIP) analog, attenuated H_2_O_2_-induced cartilage injury by upregulating chondrogenic markers and inhibiting the PI3K/AKT/NF-κB pathway, as evidenced by decreased phosphorylation of PI3K, AKT, and NF-κB (103). Similarly, urolithin A mitigated OA progression by downregulating PI3K and AKT phosphorylation, thereby blocking the NF-κB pathway in OA (120).

In a rat OA model, GLP-1R agonist liraglutide activated GLP-1R to protect chondrocytes against endoplasmic reticulum stress (ERS) and apoptosis induced by IL-1β or triglycerides (88). When using a PI3K/AKT inhibitor, the anti-apoptotic effect of GLP-1R was abolished. Activation of GLP-1R significantly inhibit the ERS induced nuclear translation of NF-κB. The chondroprotective effect of GLP-1R in OA also has been proved in a cohort study. For the knee OA patients with comorbid type 2 diabetes mellitus, GLP-1R agonists lowered the cartilage loss velocity of the medial femorotibial joint (121). However, a randomized controlled trial showed that liraglutide could not reduce knee OA pain (122).

Another study highlighted a distinct mechanism of action for liraglutide, which inhibited advanced glycation end products (AGEs)-induced production of inflammatory cytokines in primary chondrocytes. This resulted in a decrease in caspase-3 levels and a reduction in apoptotic activity. In summary, liraglutide's protective effects in OA are primarily mediated by the attenuation of apoptosis (123).

#### 4.6.3 Hypoxia-inducible factors in OA

In the human HIF family, HIF-1α and HIF-2α are mainly expressed in chondrocytes and have opposing roles in the pathogenesis of OA (70, 124). HIF-1α maintains cartilage homeostasis, while HIF-2α aggravates the pathological process of OA. Under hypoxia or activation of the NF-κB, PI3K/AKT or MAPK/MEK pathway, the expression levels of phosphorylated HIF-1α and HIF-2α are elevated in chondrocytes. The phosphorylated HIFs translocate into the nucleus where they bind to HIF-1β or HIF-2β and hypoxia response elements (HREs) to form an active transcriptional complex, which modulates downstream target genes, such as inducible nitric oxide synthase (iNOS), MMPs, and VEGF (70, 124–126).

A previous study demonstrated that the GM metabolite capsiate (CAT) was significantly lower in the OA group compared with the healthy group (127). CAT can alleviate ferroptosis in OA by decreasing the expression level of HIF-1α and oxidative stress levels and elevated solute carrier family 2 member 1 expression. A shift from HIF-1α to HIF-2α expression is considered to be one of the pathomechanisms OA (128). Hence, CAT may alleviate OA by decreasing this transformation.

#### 4.6.4 Receptor tyrosine kinases and their ligands

Receptor tyrosine kinases (RTKs) are a large class of trans-membrane receptors that regulate the intracellular signaling transmission. Many of the 20 classes of RTKs have been implicated in the development of OA owing to their effects on articular cartilage homeostasis (129).

Insulin-like growth factor-1 (IGF-1) binds to its receptor (IGF-1R) to regulate cartilage metabolism. It promotes chondrocyte proliferation, enhances matrix production, and inhibits chondrocyte apoptosis via indirectly activating the PI3K/AKT/mTOR and ERK/MAPK pathways (130). However, in IL-1β-induced apoptosis, IGF-1 inhibits chondrocytes by blocking the NF-κB, PI3K/AKT, and p38MAPK pathways (131). Yan et al. elucidated the mechanisms by which GM-induced IGF-1 regulates bone remodeling (132, 133). Colonization of germ-free mice with normal GM increased systemic and local IGF-1 levels, stimulating bone formation, resorption, remodeling, and endochondral ossification. SCFAs were proposed as potential mediators of this process. In addition, another study demonstrated the immunomodulatory effects of commensal GM on physiological bone remodeling via IGF-1 signaling (134).

**Figure 4:**
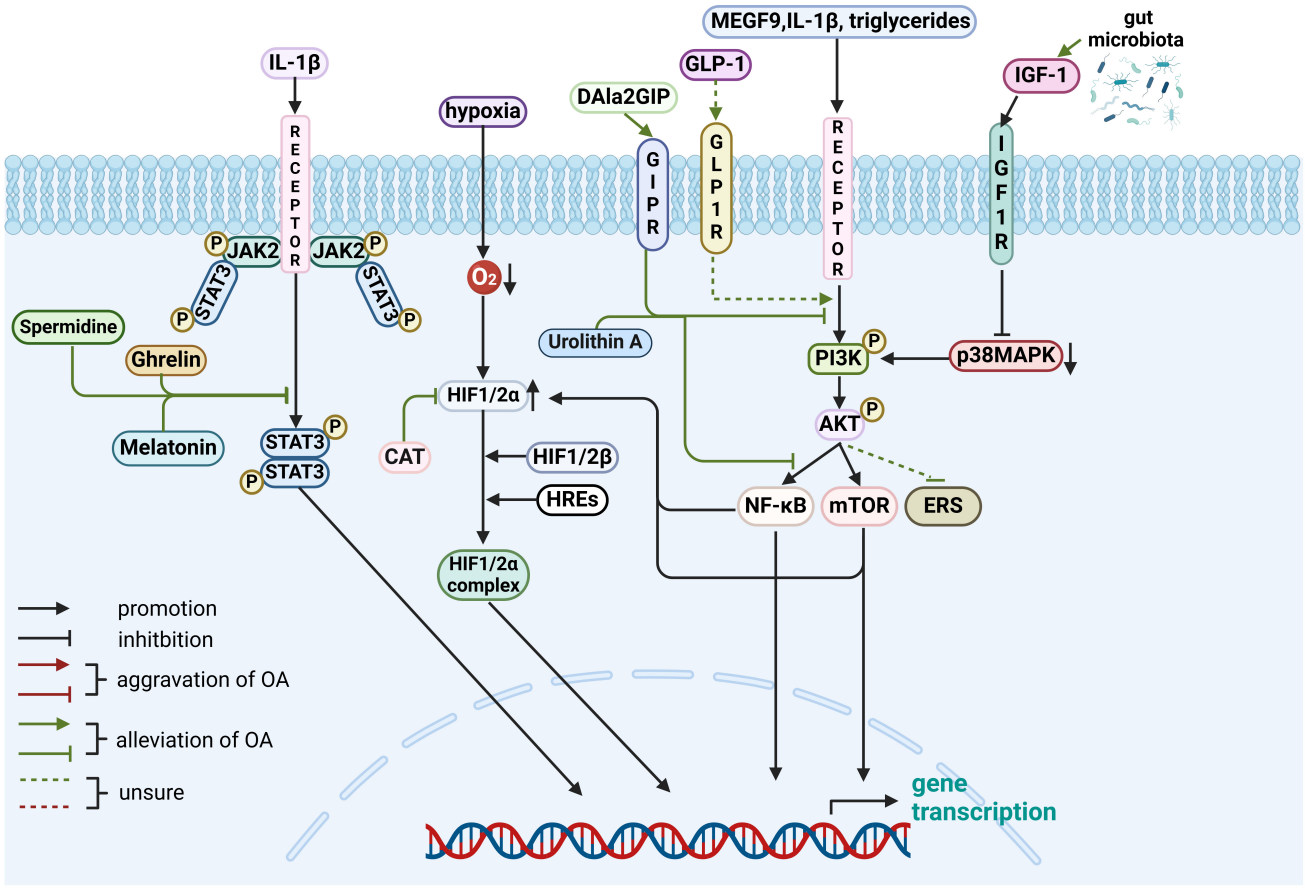
Role of gut-related factors in the pathogenesis of OA in other pathways in chondrocytes. Gut-related factors: ghrelin, melatonin, spermidine, DALa2GIP (GIP analog), urolithin A, GLP-1, CAT and GM. AKT, protein kinase B; ERS, endoplasmic reticulum stress; GLP-1, Glucagon-like peptide 1; GIPR, gastric inhibitory polypeptide receptor; HIF, Hypoxia-inducible factor; HRE, hypoxia response elements; IGF-1, Insulin-like growth factor-1; IL, interleukin; JAK2, Janus kinase 2; MAPK, mitogen-activated protein kinase; MEGF-9, multiple EGF-like-domains 9; mTOR, mammalian target of rapamycin; NF-κB, nuclear factor-kappa B; PI3K, phosphatidylinositol3-kinase; STAT3, signal transducer and activator of transcription 3. Created with BioRender.com.

The aforementioned gut-related factors associated with various pathways are presented in Figure 4. Besides, another three RTKs and their ligands will be shown in Table 1 for a brief introduction.

**Table 1:** The PDGF/PDGFR, VEGF/VEGFR, and NGF/NGFR pathways in OA and the role of gut-related factors in the pathogenesis of OA associated with these pathways.

**Signaling molecules and its receptor**	**Associated pathways**	**The effect on OA**	**GM relevant mechanism**	**References**
PDGF/PDGFR				(135–139)
PDGF-BB/PDGFR-β	/	Type H vessels synthesized, Cartilage degeneration	*Akkermansia muciniphila* promotes bone fracture healing and revascularization but unknown in OA	
Recombinant PDGF-BB	MAPK/ERK	Positive, Inhibit cartilage degradation	/	
VEGF/VEGFR	PI3K/AKT, MAPK/ERK, NF-κB	Angiogenesis, joint pain	Diet-induced steric acid stimulates VEGF production in chondrocytes	(140–145)
VEGFA, VEGFB/VEGFR1	/	Angiogenesis, joint pain	/	
VEGFA/VEGFR2		Cartilage degeneration		
VEGFC/VEGFR3		Lymph angiogenesis		
NGF/NGFR	PI3K/AKT, ERK/MAPK	Pain, Angiogenesis, promote hypertrophy and calcification of chondrocytes	*Lactobacillus acidophilus* could reduce nociceptive mediators, including NGF	(146–150)

NGF/NGFR, nerve growth factor/nerve growth factor receptor; PDGF/PDGFR, platelet-derived growth factor/platelet-derived growth factor receptor; VEGF/VEGFR, vascular endothelial growth factor/vascular endothelial growth factor receptor.

## 5 Gut microbiota-targeted therapies: signaling pathways offer insights into OA treatment

Emerging evidence highlights the role of GM on OA pathogenesis. This has prompted the development of targeted interventions aimed at modulating the gut microbiota for OA management. The following section explores these therapeutic approaches.

### 5.1 Gut-related treatments

General treatments include dietary supplements, probiotics, and prebiotics. These interventions demonstrated good efficacy controlling the progression of OA by modulating the GM and attenuating low-grade inflammation via multiple pathways (151). For example, oligofructose and chondroitin sulfate decreased the levels of inflammatory cytokines such as IL-12 and stimulate anti-inflammatory cytokines such as IL-10, possibly by modulating the GM (152, 153). Oligofructose reestablished *Bifidobacteria* and enhanced the function of various intestinal cell types and key genes associated with epithelial proliferation, water reabsorption, and barrier function as well as reduced endotoxin in circulation to protect against macrophages infiltration into the joint capsule (152). Chondroitin sulfate regulated the GM structures by decreasing *Lactobacillus* and *Proteobacteria* as well as increasing *Bacteroidetes*, which control the production of intestinal metabolites (153).

The therapeutic effect of probiotics in several OA has been validated in animal studies. *Lactobacillus rhamnosus* exerts cartilage effects in mice by inducing the expression of anabolic and chondrogenic transcription factors. Additionally, attenuation of intestinal damage and inflammation was also observed in these mice (154). *Streptococcus thermophilus* and *Lactobacillus pentosus* can protect joint cartilage by generating γ-aminobutyric acid (GABA), which can enter the joints to enhance anabolism and inhibit catabolism (155).

Prebiotics have exhibited therapeutic potential in managing OA. Quercetin, a flavonoid with prebiotic and antimicrobial properties, has demonstrated efficacy in partially restoring GM dysbiosis and reversing abnormal fecal metabolites in mice, suggesting a connection between GM and host metabolic homeostasis (156). Another study demonstrated that oral prebiotic fiber improved the outcomes of post-traumatic OA and by modulating bile acids and adrenic acid, to improve gut barrier (57).

It has been observed that exercise affects the “Gut-joint axis” in OA. Moderate exercise may remodel the GM and reduce the circulating levels of LPS, thereby inhibiting inflammation of OA (73). It also maintained the integrity of cartilage-subchondral bone unit and modified the post-traumatic OA relevant microbial shifts (157).

Novel and promising treatments for OA have emerged with a focus on the GM. Electroacupuncture, for instance, has demonstrated efficacy in alleviating knee OA pain while concurrently modulating gut microbiota composition. Specifically, it reduced pathogenic bacteria like Streptococcus and increased beneficial bacteria such as *Agathobacter* and *Bacteroide*, suggesting a potential link between gut health and OA (158). Oral administration of gold nanoparticles may exert anti-osteoarthritis effects by reshaping GM, increasing the abundance of beneficial bacteria such as *Ligilactobacillus* and *Lactobacillus*, enhancing SCFAs production to regulate macrophage polarization and cytokine production, and restoring intestinal barrier function (159).

### 5.2 Signaling pathways in the treatment of OA

*Lactobacillus acidophilus* may mitigate OA by intervening the intracellular activation of NF-κB pathway. It can reverse dysbiosis by decreasing the levels of inflammatory factors such as IL-1β, TNF-α, and NF-κB, nociceptive mediators such as VEGF and NGF, and catabolic enzymes such as MMP13 in knee joints, distal colon and spinal cord of *Lactobacillus acidophilus*-treated mice (150). The chondroprotective effects of *Lactobacillus* may be related to an increase in the butyrate-producing bacteria *Faecalibacterium*. This bacterium can modulate autophagy by increasing autolysosome formation and activating AMPK, while simultaneously restoring the PI3K/AKT pathway (24). Dietary fiber has been shown to increase the abundance of the *Bacillota* phylum, reduce gut permeability, and upregulate the expression of the sestrin2 protein in the knee, a process associated with the AMPK-mTOR signaling pathway (160).

Chondroitin sulfate has demonstrated beneficial effects in OA management. Combining chondroitin sulfate with a multi-strain probiotic formulation may potentiate these effects (161). This combination inhibits the TLR-2/4-mediated NF-κB pathway, thereby regulating cartilage metabolism. Moxibustion also influences the NF-κB pathway by activating PKA and increasing p-p65 expression, leading to reduced MMP13 and ADAMTS5 levels and increased collagen II production (162) Miya, a *Clostridium butyricum* tablet, relieved OA by maintaining GM homeostasis and subsequently upregulating AMPK and downregulating IL-1β expressions (163). This effectiveness of probiotics in the management of OA pain supported the theory of “gut-joint-brain axis” (164).

Probiotics can alleviate OA-related pain via multiple pathways, such as decreasing the expression of monocyte chemoattractant protein-1 (MCP-1), C–C chemokine receptor type 2 (CCR2), transient receptor potential cation channel subfamily V member 1 (TRPV1), and calcitonin gene-related peptide (CGRP) in the DRG; suppressing MMP, cyclooxygenase (COX)-2, MCP-1, CCR2, and pro-inflammatory cytokines expression in joint tissues; and upregulating the SCFA level or collagen II and tissue inhibitor matrix metalloproteinase 1 expression (164).

In summary, improving gut health is considered a potential novel therapeutic strategy in OA. While much of the current research is focused on *in vivo* and *in vitro* (animal models) experiments, recent clinical trials have also begun to explore the efficacy and safety of these therapeutic approaches (seen in Table 2).

**Table 2:** Clinical trials investigating gut-related interventions in OA.

**Trial number**	**Study type**	**Study population**	**Intervention**	**Dosage**	**Osteoarthritis outcomes**	**References**
-	RDBPCT	461 patients with knee OA, mainly females aged over 60 years	*Lactobacillus casei* Shirota	Skimmed milk twice daily for 6 months, totally containing 12 × 10^9^CFU	↓ VAS score, WOMAC score	(165)
					↓ Serum hs-CRP levels	
NCT04267432	RDBPCT	67 patients with knee OA, mainly females aged over 60 years	TCI633 (*Streptococcus thermophilus*)	4 TCI1633 capsules once daily for 12 weeks, totally containing 20 × 108 CFU	↔ WOMAC scores	(166)
					↔ Pain Improved: Serum sCTX-II and sCRP	
IRCT20161022030424N4	A randomized triple-blind, placebo-controlled clinical trial	70 patients with knee OA, mainly females aged around 50–59	*Saccharomyces boulardii*	Two capsules once daily for 12 weeks, totally containing 10 × 109 CFU	↓ VAS score, WOMAC score	(167)
					↓ Serum hs-CRP levels, malondialdehyde, total antioxidant capacity	
					↑ Quality of life	
-	RDBPCT	147 patients with knee OA, mainly females aged 64–75	*Streptococcus thermophilus, Bifidobacterium longu, Bifidobacterium breve, Lactobacillus*, and *Lactobacillus delbrueckii* subsp. bulgaricus	A high-potency probiotic capsule once daily for 12 weeks, containing 112 billion live bacteria	↓ WOMAC score	(168)
					↑Oxford knee score, balance scores	
					↑ Hand grip strength, gait speed (m/s)	
					↔ Knee flexion range-of-movement, resting pain, plasma 8-isoprostanes	
					↓ Plasma zonulin, plasma CRP	
					Gut changes: 1. ↓ intestinal leakiness	
					2. A robust association of balance scores with plasma markers of intestinal leakiness and inflammation	
NCT04172688	RDBPCT	54 patients (age 30–75 years) with co-morbid knee OA and obesity (BMI > 30 kg/m^2^), mainly females with a mean age of 59	Prebiotic oligofructose-enriched inulin	16 g/day oligofructose-enriched inulin for 6 months	↑ Some physical functions like 40 m fast paced test and hand grip strength	(169, 170)
					↓ Trunk fat	
					↓ The trend of knee pain	
					↔ Serum LPS and inflammatory markers	
					↑ Serum phenylalanine and tyrosine metabolism	
					Gut changes: 1. Fecal microbiota changes.	
					2. Positive correlation between Bifidobacterium abundance and 6-min walk test distance and hand grip strength	
					3. Minimal changes to fecal short-chain fatty acid	

BMI, body mass index; CFU, colony-forming unit; CRP, C-reactive protein; hs-CRP, high sensitivity C-reactive protein; LPS, lipopolysaccharide; OA, osteoarthritis; RDBPCT, randomized double-blind, placebo-controlled clinical trial; sCTX-II, serum collagen type II C-telopeptide; VAS, visual analog scale; WOMAC, Western Ontario and McMaster Universities Osteoarthritis Index.

↑, increase; ↓, decrease; ↔, no change.

## 6 Conclusion and perspectives

This review offers a novel synthesis of the emerging evidence linking gut-related factors to OA pathogenesis. To elucidate potential signaling pathways, we provide a comprehensive overview of the complex interactions, and signal transduction mechanisms, as detailed in Table 3 and Figures 1–4. Advances in technology have led to the identification of the effects of gut-related factors in human health. Gut homeostasis affects the health of joints by secreting various metabolites. Therefore, normal metabolism, strong intestinal barrier, and proper immune response are beneficial to OA development. In contrast, dysbiosis can contribute to joint impairment by altering normal immune response, disrupting the intestinal barrier, and allowing microbiota translocation. This review also highlights the potential role of gut-related factors in regulating chondrocytes through some key signaling pathways, offering insights into how intestinal factors influence cartilage health and OA progression. Although many GM-based therapies have shown good efficacy in preventing and treating OA, their mechanisms are not fully understood. This review provides a theoretical basis for future investigations.

**Table 3:** Potential gut-related factors in the pathogenesis of OA.

**Gut-related factors**	**Type**	**OA type**	**Research model**	**Role in OA**	**Involved symptoms**	**Possible mechanism**	**Pathway**	**References**
Butyrate	GM metabolite	*In vitro* OA model	Human chondrocyte culture	Positive	Pain, cartilage damage, and joint inflammation	Suppress IL-1β-induced NF-κB activation	NF-κB	(26)
		*In vitro* OA model	Mice chondrocyte culture/hypothesis			Activate AMPK with mTOR inhibition via the PI3K/AKT pathways	AMPK/mTOR	(24, 27)
LPS	Microbial structure	Obesity-related	/	Negative	Pain, cartilage damage	Bind to the TLR4 to activate TLR4/MyD88/NF-κB pathway	NF-κB	(47, 60, 74)
		*In vitro* OA model	Human chondrocyte culture	Negative		Suppress ITCH to indirectly promote JAG1	Notch	(96)
Spermidine	GM metabolite	*In vitro* OA model	Human chondrocyte culture	Positive	Cartilage damage	Suppress IL-1β-induced NF-κB activation	NF-κB	(43)
		*Vivo* and *vitro* OA model	Human chondrocyte culture/mice underwent anterior cruciate ligament transection	Positive	Cartilage damage, and joint inflammation	Enhance brahma-related gene 1 expression and suppress STAT3 phosphorylation	JAK2/STAT3	(115)
IAld, IPA	GM metabolite	*In vitro* OA model	Mice chondrocyte culture	Positive	Cartilage damage, and joint inflammation	Suppress IL-1β-induced NF-κB activation through AhR	NF-κB	(40, 41)
Melatonin	Gut processing hormone	*In vitro* OA model	Mice chondrocyte culture	Positive	Cartilage damage	Suppress IL-1β-induced NF-κB activation through up-regulating SIRT1	NF-κB	(80)
		*In vitro* OA model	Mice chondrocyte culture	Positive		Activate AMPK and Foxo3 phosphorylation	AMPK/mTOR	(104)
		*In vitro* OA model	Mice chondrocyte culture	Positive		Activating the TGF-β1/Smad2	TGF/BMP	(80)
		Monosodium iodoacetate-induced mice OA	Inject the sodium iodoacetate into the mice knee joint	Positive		Inhibit the expression of MMPs and JAK2/STAT3 signaling	JAK2/STAT3	(114)
Urolithin A	GM metabolite	*In vitro* OA model	Mice chondrocyte culture	Positive	Pain, cartilage damage, and joint inflammation	Suppress IL-1β-induced NF-κB activation	NF-κB	(84)
		*In vitro* OA model	Mice chondrocyte culture	Positive		Suppress IL-1β-induced MAPK activation	MAPK	(84)
		*In vitro* OA model	Human chondrocyte culture	Positive		Suppress PI3K/AKT/NF-κB pathway	PI3/AKT	(120)
Urolithin B	GM metabolite	*In vitro* OA model	Mice chondrocyte culture	Positive	Cartilage damage, and joint inflammation	Suppress IL-1β and TNF-α induced NF-κB activation	NF-κB	(46)
Ghrelin	Gut peptide	Posttraumatic mice OA and *vitro* OA models	Mice articular cartilage/human chondrocyte culture	Positive	Cartilage damage	Suppress IL-1β-induced NF-κB activation	NF-κB	(86)
		*In vitro* OA model	Human chondrocyte culture	Positive		Suppress the JAK2/STAT3/IRF-1 pathway	JAK2/STAT3	(113)
GLP-1	Gut peptide	*In vitro* OA model	Human chondrocyte culture	Positive	Cartilage damage, and joint inflammation	Advanced glycation end products-induced NF-κB activation	NF-κB	(90)
		*In vitro* OA model	Mice chondrocyte culture	Positive/unsure	Pain, cartilage damage, and joint inflammation	Activate GLP-1R/PI3K/AKT signaling to inhibit ERS and associated apoptosis	PI3/AKT	(88, 121, 122)
DAla2GIP	GIP analog	*In vitro* OA model	Mice chondrocyte culture	Positive	Cartilage damage	Suppress PI3K/AKT/NF-κB pathway	PI3/AKT	(171)
CAT	GM metabolite	*In vitro* OA model	Mice chondrocyte culture	Positive	Cartilage damage	Suppress HIF-1α expression and reduce ferroptosis	HIF-1α	(127)

AMPK/mTOR, AMP-activated Protein Kinase/Mammalian Target of Rapamycin; ERS, Endoplasmic Reticulum Stress; Foxo3, Forkhead Box Class O3; GIP, Gastric Inhibitory Polypeptide; GLP-1, Glucagon-like Peptide 1; GLP-1R, GLP-1 Receptor; HIF-1α, Hypoxia-inducible Factor 1 Alpha; JAK2, Janus Kinase 2; STAT3, Signal Transducer and Activator of Transcription 3; JAG1, Jagged 1; NF-κB, Nuclear Factor-kappa B; PI3K, Phosphatidylinositol 3-Kinase; AKT, Protein Kinase B; Smad2, SMAD Family Member 2; TGF, Transforming Growth Factor; BMP, Bone Morphogenic Protein.

## Glossary

ADAMTS, A Disintegrin and Metalloproteinase with Thrombospondin Motifs; Abl, Abelson; AhR, Aryl Hydrocarbon Receptor; ALK, Activin-Like Kinase; AMPK, AMP-Activated Protein Kinase; BA, Bile Acid; BMP, Bone Morphogenic Protein; CGRP, Calcitonin Gene-Related Peptide; CaMKK-β, Calcium/Calmodulin-Dependent Protein Kinase Kinase-β; CAT, Capsiate; CBF1, C-Repeat/DRE Binding Factor 1; CBF-1/suppressor of hairless/Lag1, CBF-1/Suppressor of Hairless/Lag1; CCR2, C–C Chemokine Receptor Type 2; COX, Cyclooxygenase; CyP1A1, Cytochrome P450 of Family 1, Subfamily A, Polypeptide 1; DRG, Dorsal Root Ganglion; ERS, Endoplasmic Reticulum Stress; ECM, Extracellular Matrix; ERK, Extracellular Signal-Regulated Kinase; FXR, Farnesoid X Receptor; FGF, Fibroblast Growth Factor; FOXO3a, Forkhead Box Class O 3a; GIP, Gastric Inhibitory Polypeptide; GABA, Generating γ-Aminobutyric Acid; GLP-1, Glucagon-Like Peptide 1; GPR43, G Protein-Coupled Receptor 43; Gpbar1, G-Protein-Coupled Bile Acid Receptor Gpbar1; GM, Gut Microbiota; HES, Hairy and Enhancer of Split; HREs, Hypoxia Response Elements; HIF-2α, Hypoxia-Inducible Factor-2α; IAA, Indole Acetic Acid; IAld, Indole-3-Aldehyde; IPA, Indole-3-Propionic Acid; NOS, Inducible Nitric Oxide Synthase; IKKα/IKKβ/IKKγ-NEMO, Inhibitor of Kappa B Kinase Complex; IκBα, Inhibitor of Nuclear Factor Kappa B; IGF-1, Insulin-Like Growth Factor-1; IGF-1R, Insulin-Like Growth Factor-1 Receptor; IRAK1, Interleukin-1-Receptor Associated Kinases 1; IL-1β, Interleukin-1β; ITCH, Itchy E3 Ubiquitin-Protein Ligase; IKK, IκB Kinase; JAG1, Jagged 1; JAK, Janus Kinase; LPS, Lipopolysaccharide; LKB1, Liver Kinase B1; LBP, LPS-Binding Protein; mTOR, Mammalian Target of Rapamycin; MKKKs, MAPK Kinase Kinases; MKKs, MAPK Kinases; MMPs, Metalloproteases; MAPK, Mitogen-Activated Protein Kinase; MCP-1, Monocyte Chemoattractant Protein-1; mTORC1, mTOR Complex 1; MEGF9, Multiple EGF-Like-Domains 9; MyD88, Myeloid Differentiation Factor 88; NICD, Notch Intracellular Domain; NF-κB, Nuclear Factor-Kappa B; OA, Osteoarthritis; PRRs, Pattern Recognition Receptors; PGC-1α, Peroxisome Proliferator-Activated Receptor γ Coactivator-1α; PI3K/AKT, Phosphatidylinositol 3-Kinase/Protein Kinase B; RTKs, Receptor Tyrosine Kinases; R-Smad, Receptor-Regulated Small Mothers Against Decapentaplegic; RBPJ, Recombination Signal Binding Protein for Ig Kappa J; REDD1, Regulated in Development and DNA Damage Response 1; RA, Rheumatoid Arthritis; SCFA, Short-Chain Fatty Acids; STAT, Signal Transducer and Activator of Transcription; SIRT1, Sirtuin1; SpA, Spondylarthritis; TCR, T Cell Receptor; TAK1, TGFβ-Activated Kinase-1; TACE, TNF-α Converting Enzyme; TLR, Toll-Like Receptor; TGF-β, Transforming Growth Factor-Beta; TRPV1, Transient Receptor Potential Cation Channel Subfamily V Member 1; Trp, Tryptophan; TSC2, Tuberous Sclerosis Complex 2; TNFR, Tumor Necrosis Factor Receptor; TRAF6, Tumor Necrosis Factor Receptor-Related Factor 6; TNF-α, Tumor Necrosis Factor-α; Ub, Ubiquitin; VEGF, Vascular Endothelial Growth Factor; ZO-1, Zonula Occludens-1.
